# FK506 induces lung lymphatic endothelial cell senescence and downregulates LYVE-1 expression, with associated decreased hyaluronan uptake

**DOI:** 10.1186/s10020-020-00204-z

**Published:** 2020-07-31

**Authors:** Shikshya Shrestha, Woohyun Cho, Benjamin Stump, Jewel Imani, Anthony M. Lamattina, Pierce H. Louis, James Pazzanese, Ivan O. Rosas, Gary Visner, Mark A. Perrella, Souheil El-Chemaly

**Affiliations:** 1 Division of Pulmonary and Critical Care Medicine, Brigham and Women’s Hospital, Harvard Medical School, Boston, MA 02115 USA; 2 Present Address: Division of Pulmonology, Allergy and Critical Care Medicine, Department of Internal Medicine, Pusan National University Yangsan Hospital, Yangsan, Republic of Korea; 3 Deparmtent of Pediatrics, Boston Children Hospital, Harvard Medical School, Boston, MA USA

**Keywords:** Lung lymphatic, Endothelial cells, LYVE-1, TERT, Senescence, Fk506, Hyaluronan

## Abstract

**Background:**

Therapeutic lymphangiogenesis in an orthotopic lung transplant model has been shown to improve acute allograft rejection that is mediated at least in part through hyaluronan drainage. Lymphatic vessel endothelial hyaluronan receptor (LYVE-1) expressed on the surface of lymphatic endothelial cells plays important roles in hyaluronan uptake. The impact of current immunosuppressive therapies on lung lymphatic endothelial cells is largely unknown. We tested the hypothesis that FK506, the most commonly used immunosuppressant after lung transplantation, induces lung lymphatic endothelial cell dysfunction.

**Methods:**

Lung lymphatic endothelial cells were cultured in vitro and treated with FK506. Telomerase activity was measured using the TRAP assay. Protein expression of LYVE-1 and senescence markers p21 and β-galactosidase was assessed with western blotting. Matrigel tubulation assay were used to investigate the effects of FK506 on TNF-α-induced lymphangiogenesis. Dual luciferase reporter assay was used to confirm NFAT-dependent transcriptional regulation of LYVE-1. Flow cytometry was used to examine the effects of FK506 on LYVE-1 in precision-cut-lung-slices ex vivo and on hyaluronan uptake in vitro*.*

**Results:**

In vitro*,* FK506 downregulated telomerase reverse transcriptase expression, resulting in decreased telomerase activity and subsequent induction of p21 expression and cell senescence. Treatment with FK506 decreased LYVE-1 mRNA and protein levels and resulted in decreased LEC HA uptake. Similar result showing reduction of LYVE-1 expression when treated with FK506 was observed ex vivo*.* We identified a putative NFAT binding site on the LYVE-1 promoter and cloned this region of the promoter in a luciferase-based reporter construct. We showed that this NFAT binding site regulates LYVE-1 transcription, and mutation of this binding site blunted FK506-dependent downregulation of LYVE-1 promoter-dependent transcription. Finally, FK506-treated lymphatic endothelial cells show a blunted response to TNF-α-mediated lymphangiogenesis.

**Conclusion:**

FK506 alters lymphatic endothelial cell molecular characteristics and causes lymphatic endothelial cell dysfunction in vitro and ex vivo. These effects of FK506 on lymphatic endothelial cell may impair the ability of the transplanted lung to drain hyaluronan macromolecules in vivo*.* The implications of our findings on the long-term health of lung allografts merit more investigation.

## Background

Lung transplant carries the worst outcome of any solid organ transplant with a 60% 5-year survival (Yusen et al., 2010; Valapour et al., 2018). Acute rejection represents the single most important risk factor for chronic lung allograft dysfunction (CLAD), a major contributing factor to poor long-term outcomes (Chambers et al., 2017; Boehler & Estenne, 2003).

The lymphatic vasculature plays important roles in lung homeostasis, by clearing immune cells and draining interstitial fluid (Alitalo, 2011; Cui et al., 2017; Stump et al., 2017). The role of the lymphatic vasculature in acute and chronic lung rejection is only beginning to be unraveled. Tissue examination has shown no change in lymphatic vessel density in CLAD compared to normal lung (Traxler et al., 2017). However, in acute rejection, there seems to be an increase in lymphatic vessel density (Dashkevich et al., 2010). The accumulation of short-fragment Hyaluronic Acid (HA) has been linked to organ injury, graft dysfunction, and transplant rejection; HA accumulation has been linked with HA forming complexes with various membrane receptors such as CD44, LYVE-1 and TSG-6 (Cui et al., 2015; Jiang et al., 2005; Jiang et al., 2007; Todd et al., 2014; Courtwright et al., 2019; Ouasti et al., 2012; Jackson, 2004). Lymphatics are responsible for 85% of HA catabolism and clearance, which amounts to several grams/day in humans (Ouasti et al., 2012; Jackson, 2004). We have recently shown in a mouse model of orthotopic lung transplant that inducing lymphangiogenesis results in an improvement in acute lung allograft rejection, which is associated with a decrease in short-fragment HA (Cui et al., 2015). This occurs primarily through the endocytic lymphatic vessel endothelial HA receptor (LYVE-1), on the surface of lymphatic endothelial cells (LEC) (Prevo et al., 2001).

Tacrolimus (FK506) is a mainstay of immune-suppression in lung (Valapour et al., 2018; Penninga et al., 2013) and other solid organ transplants (Scalea et al., 2016). FK506 binds FK506 binding protein 12 (FKBP-12) and inactivates calcineurin, which blocks dephosphorylation of the nuclear factor of activated T-lymphocyte (NFAT) and subsequent translocation of NFAT into the nucleus (Jain et al., 1993; Organ et al., 2017; Srikanth & Gwack, 2013). NFAT inhibition results in decreased production of pro-inflammatory cytokines (Wiederrecht et al., 1993; Li et al., 2011).

To date, the effects of FK506 on lymphatic endothelial cells have been poorly studied, with one study suggesting that in obesity, inhibition of inflammation with FK506 results in improved lymphatic function (Torrisi et al., 2016). However, to our knowledge, no studies have examined the direct effects of FK506 on LEC. The goal of our studies was to investigate the potential effects of FK506 on lung lymphatic endothelial cells. Our data show that exposure to FK506 results in LEC dysfunction by inducing senescence and decreased LYVE-1 expression, and subsequent decrease in HA uptake.

## Methods

### Additional information in supplemental information

#### Cell culture and treatment

Primary human lung lymphatic endothelial cells (LEC) (Lonza, Cat#CC-2527, Walkersville, MD) were cultured in microvascular cell culture media with supplements (Cell Applications Inc., San Diego, CA). All experiments were done with LEC between passages 3 to 5. FK506 (Sigma, Cat#F4679, St. Louis, MO) and Cyclosporin A (Sigma, Cat# 30024) were reconstituted with DMSO and used at indicated concentrations. At the 48 h time point, FK506 did not affect LYVE-1 protein levels (Supplementary Figure 1). All protein analysis experiments were then conducted at 72 h post-treatment.

#### Telomerase activity

Equal amounts of protein from FK506- or DMSO-treated LECs were used to measure telomerase activity using telomeric repeat amplification protocol (TRAP) assay (TRAPeze Telomerase Detection kit: Millipore; Billerica, MA) per manufacturer’s instruction and as we have previously described (El-Chemaly et al., 2011).

#### Luciferase reporter assay

A renilla-luciferase dual reporter assay was carried out to examine the LYVE-1 promoter activity of promoter (P)-124/+ 124 wildtype (NFATc binding site – ttttcc) and P-124/+ 125 mutant (NFATc binding site – ttgtcc) constructs. Reverse transfection was carried out on a total of 20,000 HEK-293 T cells using X-tremeGENE HP DNA Transfection Reagent (Sigma). Cells were transfected with 5 ng of pRL_CMV vector (internal control, Promega, Cat# E2261) and 95 ng of P-124/+ 214 (wildtype or mutant) construct or pGL3_Basic vector (negative control). Cells were treated with 15 ng/mL of FK506 at 6 h post-transfection, and luciferase activity was tested 24 h after transfection using Dual-Glo Luciferase Assay System kit (Promega) as per manufacturer’s protocol. Biotek Synergy HT microplate reader (BioTek, VT) with Biotek Gen5.1.1 microplate data collection software was used for luciferase luminescence detection. Each transfection was carried out in triplicates in 4 independent experiments.

#### Matrigel tubulation

Matrigel tubulation assays were conducted as we previously described (Stump et al., 2019). Briefly, LEC were treated with FK506 or with DMSO for 24 h, followed by seeding on growth factor reduced Matrigel (Corning, Corning, NY). Cells were then incubated with tumor necrosis factor (TNF-α) (100 ng/ml) for 16 h. Cells were then incubated with Calcein-AM (8 μg/ml) for 30 min. Five to ten non-overlapping 40X fluorescent images were obtained and analyzed using the ImageJ Angiogenesis Analyzer plug-in (Carpentier, 2012).

#### Hyaluronan uptake assay

The assay was performed with modifications of previously described methods (Prevo et al., 2001). Briefly, LEC were seeded in 6-well plates at a density of 50,000 cells per well. Cells were starved for 6 h and treated with DMSO or FK506 (15 ng/ml) the next day for 72 h. For control experiment, cells were treated with isotype control (10 μg/mL; R&D Systems, Minneapolis, MA, Cat#MAB002) and LYVE-1 antibody (10 μg/mL; R&D System, Cat#MAB20892) for 16 h. After incubation with each treatment, cells were treated with 1000 μg/ml FITC-labelled HA (> 1000 kDa, Matexcel, Bohemia, NY, Cat#NAT-167) for 5 h. To analyze the cellular uptake of HA by live LEC cells, 7AAD staining was performed and FITC-positive and 7AAD-negative cells were analyzed by flow cytometry (BD FACSCanto II, BD-Biosciences, San Jose, CA). FITC-positive cells were also observed using an Olympus FluoView FV-10i confocal laser-scanning microscope (Olympus, Tokyo, Japan) on chambered coverglass system (ThermoFisher Scientific, MA, Cat#155382).

#### Precision Cut Lung Slices (PCLS)

PCLS from C57BL/6-Tg (Prox1-tdTomato)12Nrud/J mice (Jackson Laboratory, Bar Harbor, ME) were obtained as previously described (Rosner et al., 2014). Briefly, after tracheotomy, lungs were inflated with 1% low-melting-point agarose in Hanks’ balanced salt solution (HBSS), and agarose allowed to gel in cold HBSS. Lungs were then sliced into 200 μm-thick slices using a tissue slicer (VF-300; Precisionary Instruments, Greenville, NC). Slices were then cryopreserved in 10% DMSO diluted in Dulbecco’s modified Eagle/F-12 medium and stored in liquid nitrogen. Cryovials were thawed at 37 **°**Cin water bath and recovered for 16 h in complete DMEM medium. Slices were treated with DMSO or 15 ng/mL FK506 in 1% FBS media for 7 days, while replacing fresh media every 48 h.

#### Flow Cytometry

After treatment, lung cells were isolated by digesting PCLS slices in media containing collagenase type 4 (300 U/mL; Cat#LS004188, Worthington Biochemical Corp.), hyalurodinase (1000 U/mL; Cat#H3506, Sigma), and DNase I (50 U/mL; Cat#D4527, Sigma) for an hour at 37 °C. Cells were then washed twice with cold FACS buffer (PBS with 1% BSA), and incubated with purified rat anti-mouse CD16/CD32 Fc block solution (Cat# 553142; BD Biosciences) for 20 min at 4 °C, followed by incubation with anti-LYVE1 monoclonal antibody conjugated with PE-Cyanine7 (Cat# 25–0443-80, Thermo Fisher Scientific) for 30 min at 4 °C. Lastly, cells were washed twice with FACS buffer and subjected to flow cytometry analysis.

### Statistical analysis

All experimental data are presented as the mean ± SEM. For analysis of western blot and q-PCR data, one-way repeated measure ANOVAs (RM ANOVA) (within-subjects factor: treatment) were run, followed by Tukey’s post-hoc test to evaluate treatment effect according to dose of FK506 (10 or 15 ng/ml). To compare immunofluorescent intensity values between positive control and FK506 (15 ng/ml)-treated cells, two-tailed Student’s *t*-tests were conducted. Telomerase activity in control and FK506 (15 ng/ml)-treated cells was compared using a paired, two-tailed Student’s *t*-test. A *P* value of less than 0.05 was considered significant.

## Results

### FK506 results in decreased nuclear NFAT

FK506 inactivates calcineurin, which blocks the dephosphorylation of NFAT, a necessary step for its nuclear translocation and subsequent transcription activation. To confirm that FK506 blocks NFAT nuclear translocation in lung LEC, we treated LEC with FK506 and showed with cell fractionation that treatment resulted, as expected, in decreased NFAT nuclear translocation (Supplementary Figure 2).

### FK506 downregulates TERT and decreases telomerase activity

**Fig. 1:**
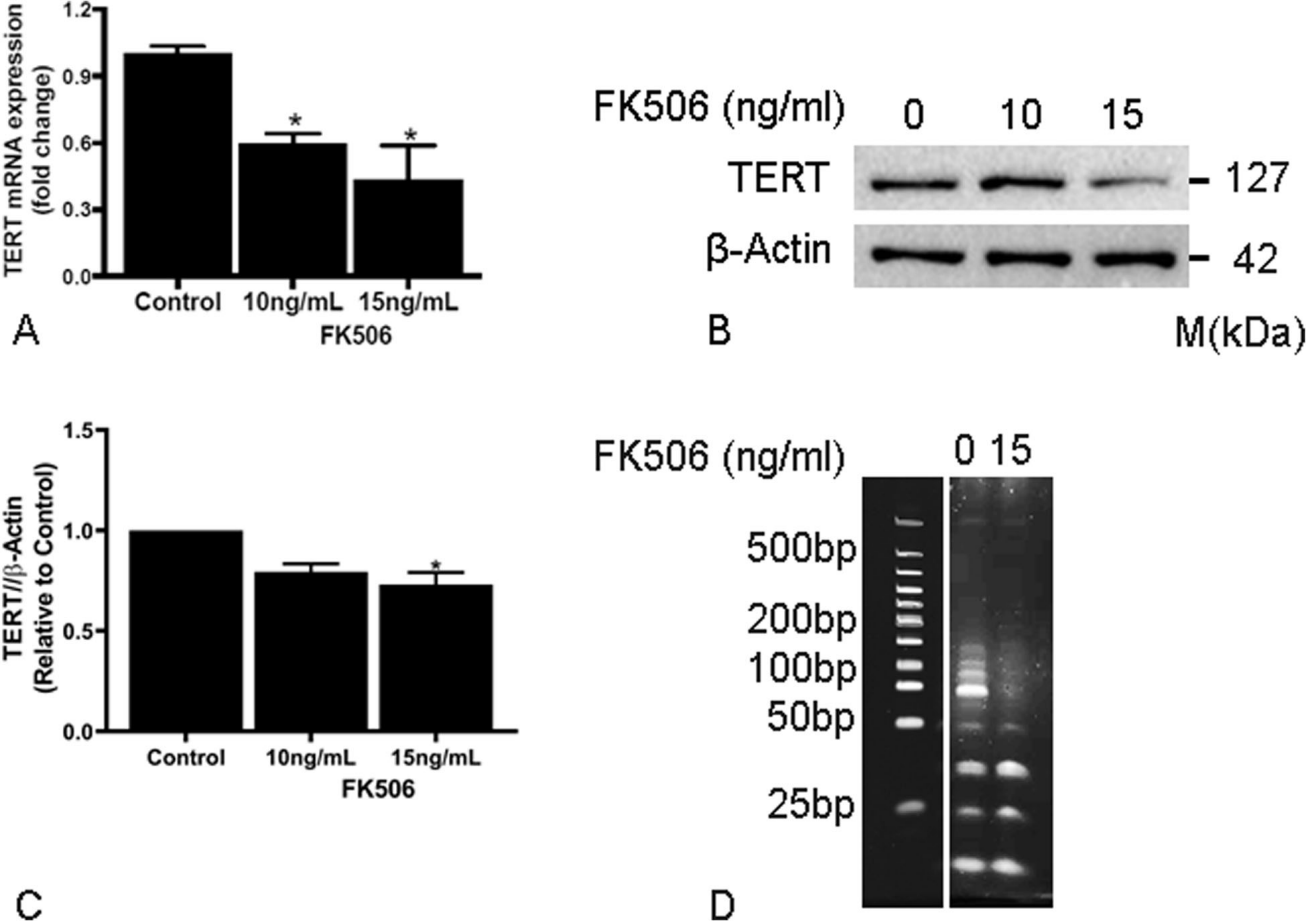
FK506 decreases TERT mRNA and protein levels with resulting decrease in telomerase activity. Lung lymphatic endothelial cells were treated for 48 (**a, d**) or 72 h (**b**). (**a**) Real-time PCR analysis of TERT mRNA in control and FK506-treated lung lymphatic endothelial cells. Results were expressed as the fold change compared to control. (**b**) Whole cell lysates were analyzed by Western blotting with antibodies against NFAT and TERT. (**c**) Ratio of TERT to β-actin density was expressed as fold change compared to control. Data represent mean ± SEM of 3 independent experiments (*p < 0.05) by one-way ANOVA; each independent experiment for western blotting consisted of one technical replicate. (**d**) Treatment with FK506 (15 ng/ml) resulted in decreased telomerase activity as assayed by the TRAP assay (*p* < 0.05). Experiment was repeated three times

Telomerase reverse transcriptase (TERT) is a key enzyme involved in telomere maintenance (Cong et al., 2002; Bernadotte et al., 2016). FK506 is a known inhibitor of calcineurin activation and the NFAT signaling pathway, and TERT is a known NFAT transcriptional target (Chebel et al., 2009). We first examined the effects of FK506 on TERT RNA expression in LEC. We found a ~ 60% decrease in TERT mRNA levels after treatment with FK506 (Fig. 1a). Similarly, treatment with another calcineurin inhibitor, Cyclosporin A significantly reduced TERT mRNA expression (Supplementary Figure 3A). Furthermore, Western blot analysis showed that treatment with FK506 (15 ng/ml) results in a decrease in TERT protein levels as well (Fig. 1b, c). To evaluate the effects of downregulation of TERT on telomerase activity, a TRAP assay was performed. We found a consistent and significant decrease in telomerase activity in LEC treated with 15 ng/ml FK506 (Fig. 1d).

### FK506 induces lung LEC senescence in vitro

**Fig. 2:**
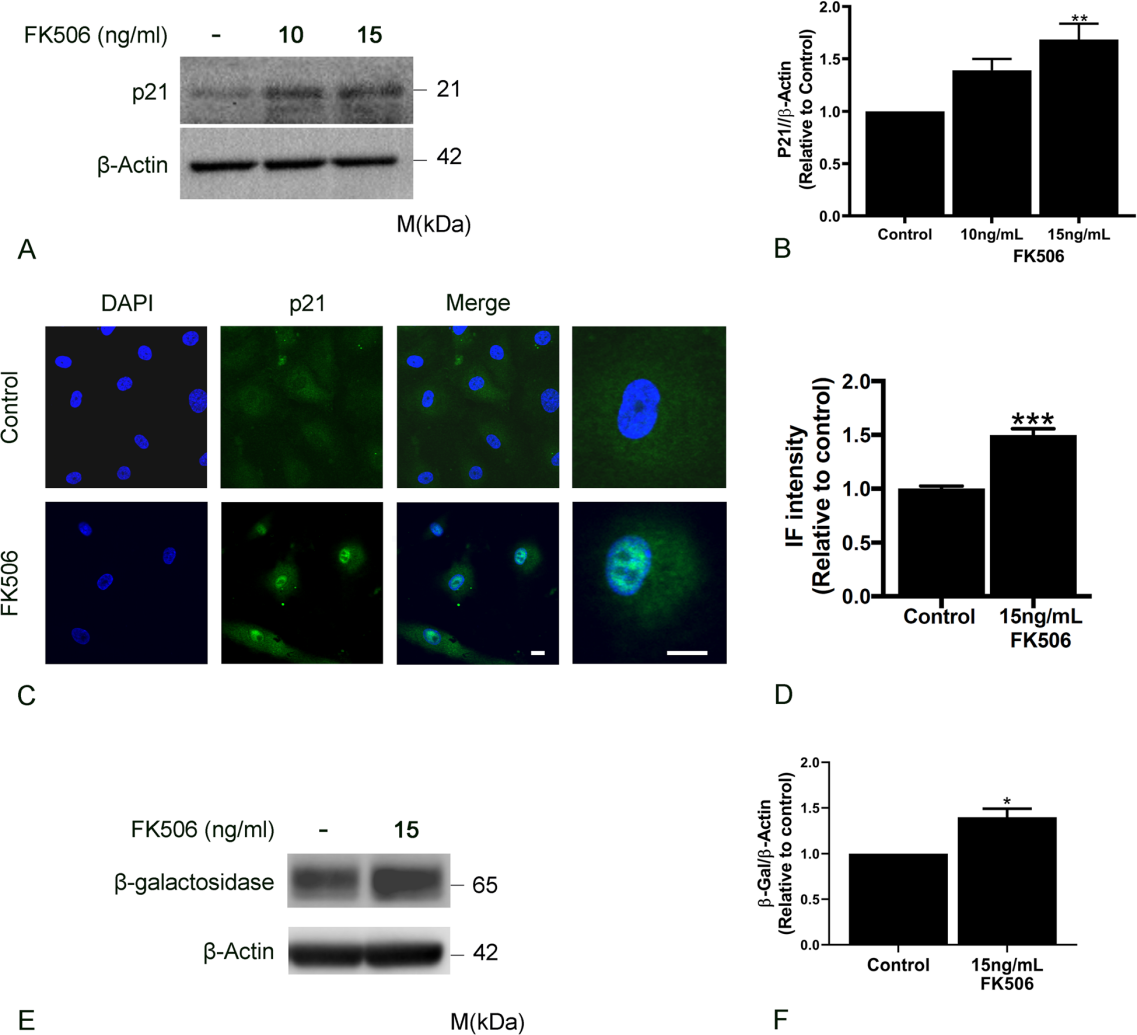
FK506 induces Lymphatic endothelial cell senescence. Lung lymphatic endothelial cells were treated with FK506 (15 ng/ml) for 72 h. Whole cell lysates were analyzed by Western blotting with antibodies against p21(**a**) and β-galactosidase (**e**). Ratio of p21 (**b**) and β-galactosidase (**f**) to β-actin density was expressed as fold-change relative to control. Data represent mean ± SEM of three independent experiments (one technical replicate each) * *p* < 0.05 by one-way ANOVA. Representative images of immunofluorescent staining of single and merged images of p21 (green) and 4′,6-diamidino-2-phenulindole (DAPI; blue) at low and high magnification (far right panel) with and without treatment with FK506 (15 ng/ml) for 72 h (**c**). Random images (9–12 images) were obtained and nuclear intensity of p21 staining was measured using Image J software (**d**). Data represent mean ± SEM of one experiment (**p < 0.01). Results were reproduced in 3 independent experiments (Size bar: 40 μm)

Telomere dysfunction has been shown to induce p21 expression and cell cycle arrest (Aix et al., 2016). We hypothesized that FK506-dependent decrease in TERT and telomerase activity, would similarly lead to p21 expression and LEC senescence. Indeed, we found that treatment with FK506 (15 ng/ml) resulted in an increase in p21 protein expression (Fig. 2a, b). These results were also confirmed with immunostaining and confocal microscopy showing an increase in p21 nuclear intensity in LEC treated with FK506 (15 ng/ml) (Fig. 2c, d). In addition, FK506 resulted in increased expression of another senescence marker β-galactosidase (Fig. 2e, f) (Herranz & Gil, 2018). Taken together these data demonstrate that exposure to FK506 leads to downregulation of TERT and LEC senescence.

### FK506 downregulates LYVE-1 mRNA and protein expression in LEC

**Fig. 3:**
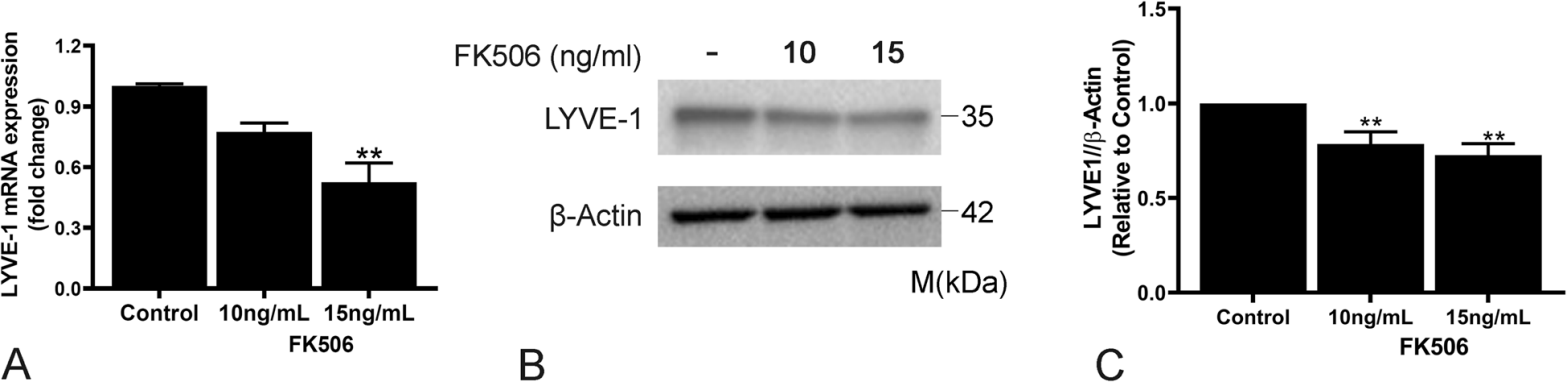
FK506 induces LYVE-1 downregulation. **a**) Real-time PCR analysis of LYVE-1 mRNA in control and FK506-treated (48 h) lung lymphatic endothelial cells. Results were expressed as the fold change compared to control. Graphs represent the mean ± SE from three independent experiments (** < 0.01 one-way ANOVA). Whole lysates of lung lymphatic endothelial cells were subjected to Western blot analysis with antibodies against LYVE-1 and β-actin (**b**). Ratio of LYVE-1 (**c**) to β-actin density was expressed as fold-change relative to control. Data represent mean ± SEM of three independent experiments, consisting of one technical replicate each. **p < 0.01 by one-way ANOVA

Hyaluronan is a critical mediator of lung injury and repair (Jiang et al., 2007) and has been implicated in acute and chronic lung allograft rejection (Cui et al., 2015; Todd et al., 2014). LYVE-1 is a critical receptor for HA on the surface of LEC. Little is known about LYVE-1 transcriptional regulation. We examined the effects of FK506 on LYVE-1 expression and found that treatment with FK506 resulted in a ~ 50% reduction in LYVE-1 mRNA (Fig. 3a). Consistent with this decrease in mRNA levels, LYVE-1 protein expression was also decreased (Fig. 3b, c). The significant (~ 60%) decrease in LYVE-1 mRNA expression was also observed after treatment with cyclosporin A -another calcineurin inhibitor- (Supplementary Figure 3B). We also examined the effect of FK506 treatment on additional lymphatic markers (PDPN and PROX1), which showed no significant difference compared to control treatment (Supplementary Figure 4A-B).

### Regulation of LYVE-1 expression by NFAT

**Fig. 4:**
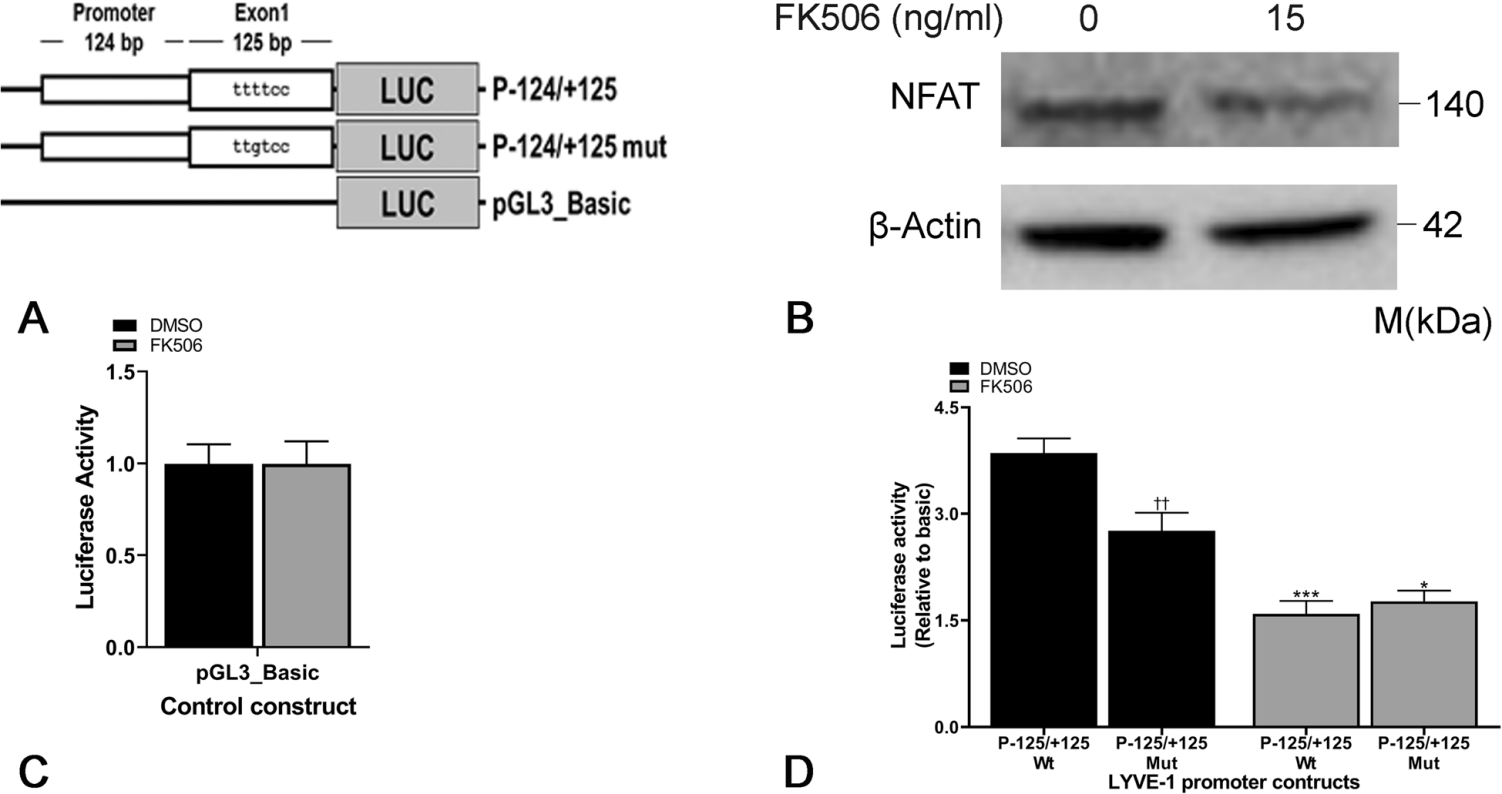
Luciferase activity of LYVE-1 promoter expression constructs. Schema of pGL3_Basic vector, and wildtype and mutant LYVE-1 luciferase promoter constructs transfected into HEK-293 T cells (**a**). Endogenous NFAT expression in HEK-293 T cells (**b**). Luciferase activity of vehicle (DMSO) and FK506 treated HEK-293 T cells transfected with (**c**) pGL3_Basic vector and (**d**) wildtype and mutant LYVE-1 promoter constructs. Each luciferase data is normalized to internal Renilla control. Data for LYVE-1 promoter constructs are presented relative to the pGL3_Basic vector luciferase activity. Average (± standard deviations as error bars) of four independent experiments are presented; each independent experiment for western blotting consisted of three technical replicates. *** *p* < 0.0001 (between P-125/+ 125 wildtype DMSO vs. FK506), * *p* < 0.05 (between P-125/+ 125 mutant DMSO vs. FK506), †† *p* < 0.001 (between P-125/+ 125 wildtype DMSO vs. P-125/+ 125 mutant DMSO) by 2-way ANOVA (Tukey’s multiple comparison) test

To confirm that NFAT transcriptionally regulates LYVE-1 expression, we examined the promoter sequence of LYVE-1 gene upstream of the transcription start site for the presence of NFATc binding sequence Matys et al., 2006). Schema of pGL3 Basic vector and LYVE-1 wildtype and mutant promoter constructs are presented in Fig. 4a. These constructs were transiently transfected in HEK-293 T cells, which express endogenous NFAT (Fig. 4b). As expected, no difference in luciferase activity was observed between DMSO and FK506 treated cells transfected with pGL3_Basic vector alone with no NFAT binding sites (Fig. 4c).

P-124/+ 125 construct showed significantly higher luciferase activity (four-fold) compared to pGL3_Basic vector alone (*p* < 0.01). Treatment with FK506 resulted in a reduction in luciferase activity, which was not observed in pGL3_Basic vector with no NFAT binding sites (Fig. 4d), suggesting the role of NFAT in LYVE-1 transcriptional regulation. The P-124/+ 125 mutant construct, with mutated NFAT binding sequence, showed overall reduced luciferase activity (*p* < 0.01), and a significant reduction (*p* < 0.05) in the response to FK506 compared to the wildtype construct (Fig. 4d).

### FK506 inhibits TNF-α-induced lymphangiogenesis in vitro

**Fig. 5:**
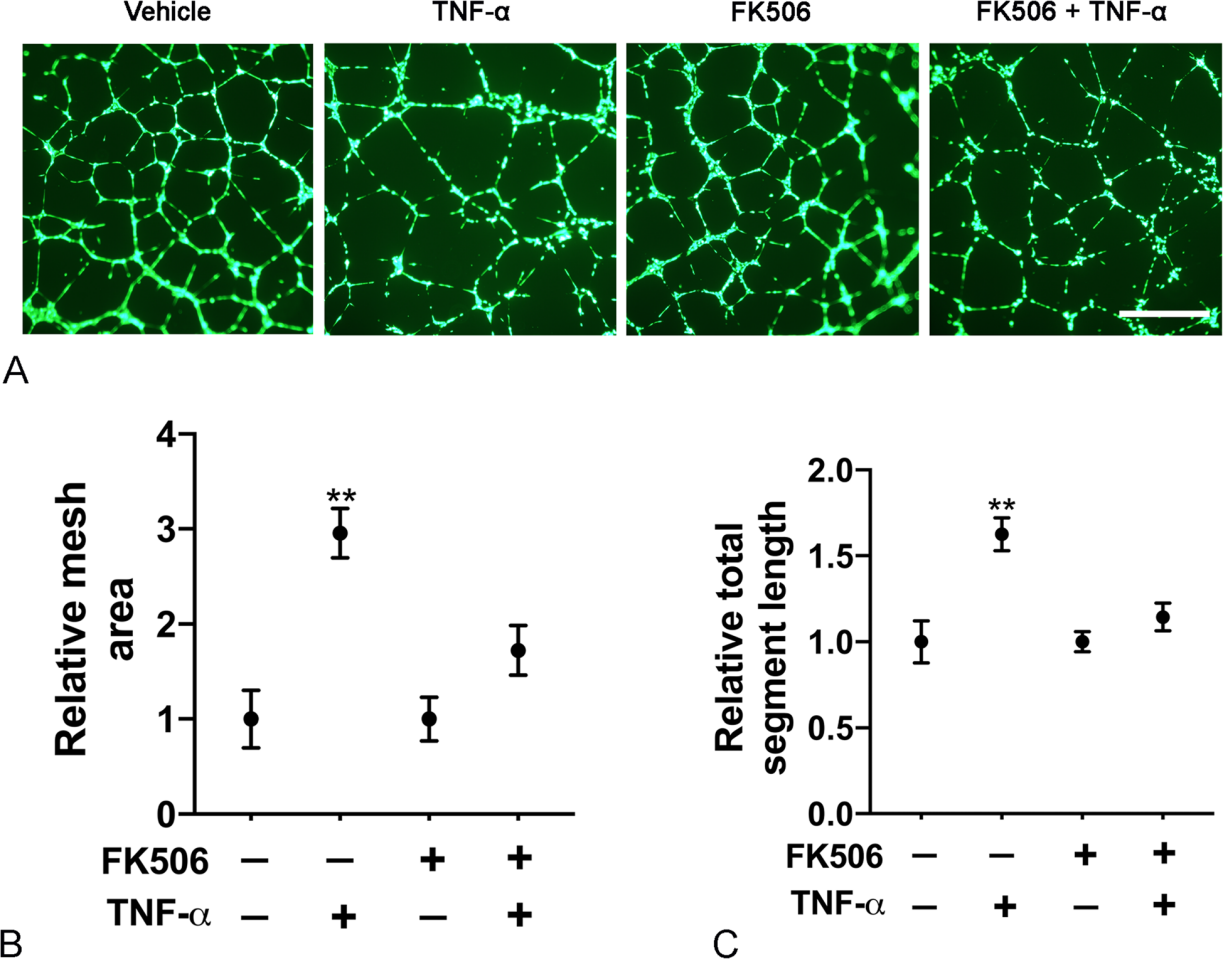
FK506 inhibits TNF-α-induced lymphangiogenesis. Lung lymphatic endothelial cells were grown in 6 well dishes and treated or not with FK506 for 48 h (15 ng/ml). Cells were then placed on Matrigel and treated as indicated with or without TNF-α (100 ng/ml) or FK506 (15 ng/ml) for 16 h. After Calcein-AM labeling, random 4X images were taken and representative images of lymphangiogenesis in different treatment groups after Calcein-AM labeling are presented (**a**) (Size bar 200 μm). Mesh area (**b**) and total segment length (**c**) were analyzed with ImageJ Angiogenesis Analyzer plugin. Results are expressed as mean ± SEM of one experiment. *** *P* < 0.001 by one-way ANOVA. Experiment was repeated once

TNF-α has been shown to induce lymphangiogenesis in vitro and in vivo (Baluk et al., 2009; Ji et al., 2014). NFAT is a known downstream effector of TNF-α (Yarilina et al., 2011). We hypothesized that the presence of FK506 would inhibit the pro-lymphangiogeneic activity of TNF-α. Indeed, in a Matrigel tubulation assay, TNF-α resulted in ~ 3-fold increase in LEC mesh area. Pre-treatment with FK506 inhibited TNF-α-induced lymphangiogenesis (Fig. 5). These data suggest that exposure to FK506 impairs the ability of lung lymphatic endothelial cells to respond to inflammatory stimuli.

### FK506 reduces HA uptake in vitro

**Fig. 6:**
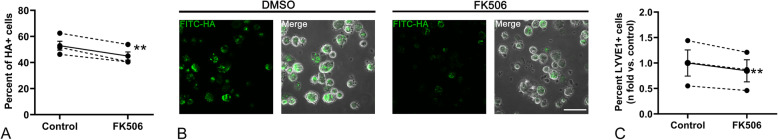
FK506 reduces HA uptake in vitro and LYVE-1 expression ex-vivo in PCLS. LEC were plated in 6-well plates and treated with vehicle or FK506 (15 ng/mL) for 72 h. Cells were then incubated in media containing 1000 μg/mL of FITC-HA for 5 h. Percentage of FITC-positive cells (**a**) were analyzed by flow cytometry. Representative images of immunofluorescent staining of single and merged images of HA (green) and DIC without and with treatment with FK506 (15 ng/ml) for 72 h (**b**) (Size bar: 50 μm). Precision cut lung slices obtained from dt-Tomato Prox1 mice, treated with FK506 (15 ng/mL) or DMSO for 7 days, followed by digestion and cell isolation. Percent LYVE1+/dt-Tomato-Prox1+ cells (**c**) were analyzed by flow cytometry. Results for flow cytometry are expressed as mean ± SEM of 3 experiments. Dotted line represents each data point and solid line represents mean of 3 experiments

Our data indicated reduced LYVE-1 gene and protein expression when treated with FK506. To assess the effect of FK506-mediated downregulation of LYVE-1 in HA uptake by LEC, LEC treated with or without FK506 were incubated with FITC-HA and analyzed by flow cytometry. FK506 treatment resulted in significant difference in relative FITC-HA positive cells compared to DMSO treatment, resulting in overall 17.5% reduction in percent of FITC-HA positive cells (*p* < 0.05; Fig. 6a). To further show that HA uptake is LYVE-1 dependent, LEC treated with isotype control or LYVE-1 function blocking monoclonal antibodies were incubated with FITC-HA and analyzed by flow cytometry. Inhibition of LYVE-1 by the function blocking antibodies resulted in ~ 60% reduction in percent FITC-HA positive cells compared to isotype control (*p* < 0.05; Supplementary Figure 5A). Additionally, we showed that FK506 treatment resulted in no significant change in CD44 expression suggesting that FITC-HA uptake in vitro is CD44–independent (Supplementary Figure 5B). Confocal immunofluorescence microscopy showed a similar decrease in FITC-uptake in FK506-treated LEC (Fig. 6b).

### FK506 reduces LYVE-1 expression ex-vivo in PCLS

Prox-1 is a transcription factor necessary and sufficient for lymphatic lineage (Oliver & Srinivasan, 2010). Precision-cut lung slices from dt-Tomato Prox-1 mice lungs were treated in 1% FBS and FK506 (15 ng/ml) or DMSO for 7 days. Tissues were then digested, and LYVE-1 expression was assessed using flow cytometry (LYVE1+/dt-Tomato-Prox1+ cells). FK506 treatment reduced LYVE-1 expression in mPCLS cells by 16.4% (Fig. 6c). This reduction in LYVE-1 expression ex vivo was similar to the reduction in HA uptake observed with FK506 treatment in vitro*.*

## Discussion

Here we show the effects of FK506, the main immunosuppressive agent used in lung transplantation, on lung lymphatic endothelial cells. Our data demonstrate that FK506 1) decreased TERT expression and telomerase activity, which was associated with increase in LEC senescence; 2) decreased LYVE-1 expression and LEC HA-uptake and 3) inhibited TNF-α-induced lymphangiogenesis.

Calcineurin inhibitors, including FK506, are currently the mainstay of immunosuppression in lung transplantation, with important effects on T-cell function and prevention of allograft rejection (Penninga et al., 2013; Valapour et al., 2015; Valapour et al., 2017). Our data showing that FK506 decreases TERT expression are in line with previous publications showing similar effects of FK506 in breast cancer (MCF7) and immortalized T-cells (jurkat cell lines) (Chebel et al., 2009). Here, we also show that this decrease in TERT expression leads directly to decreased telomerase activity and cell senescence in primary lung lymphatic endothelial cells as shown by a marked increase in P21 expression. Intriguingly, aged lymphatic vessels have a decreased ability to transport bacteria from tissues to draining lymph nodes, due in part to increased lymphatic vessel permeability (Zolla et al., 2015).

LYVE-1 plays important roles in HA uptake and subsequent degradation (Cui et al., 2015; Prevo et al., 2001). Binding of HA to LYVE-1 is dependent on LYVE-1 receptor expression and is threshold-dependent (Lawrance et al., 2016). Further, LYVE-1 serves as a docking receptor for HA and allows immune cell trafficking to the lymphatics (Jackson, 2019) and lack of the LYVE-1 receptor prevents leukocyte trafficking and exacerbates chronic inflammation (Vieira et al., 2018). Here we show that NFAT regulates LYVE-1 transcription and that the presence of an NFAT-binding site on the LYVE-1 promoter is critical for FK506 downregulation of LYVE-1 transactivation in vitro. In addition, FK506 downregulated LYVE-1 expression in PCLS ex vivo. These changes represent direct effects of FK506 on LYVE-1 expression, since in the absence of TNF-α, FK506 does not have any effects on lymphangiogenesis (data not shown) (Gardenier et al., 2017). Additionally, the downregulation of LYVE-1 is coupled with decreased LEC-HA-uptake in vitro.

In addition to LYVE-1, CD44 is another important receptor of HA with close homology to LYVE-1 (Jackson, 2004). However, FK506 treatment did not result in significant change in CD44 mRNA expression, suggesting the effect on HA uptake in vitro is likely CD44-independent. This was further corroborated by inhibiting LYVE-1 with function blocking antibody, which significantly reduced the HA uptake. Additionally, Tumor necrosis factors stimulated gene-6 (TSG-6) is another important HA binding protein (Lawrance et al., 2016; Lesley et al., 2004; Lauer et al., 2013; Coulson-Thomas et al., 2016; Jadin et al., 2014). Unlike CD44 and LYVE-1, TSG-6 is a soluble protein, mostly stored in the secretory granules of neutrophils and mast cells, which is released in response to proinflammatory signals (Lauer et al., 2013). TSG-6 has been shown to have HA interacting link module similar to, but with higher affinity to bind HA than, CD44 (Lesley et al., 2004). TSG-6 linked HA has been shown to facilitate increased binding to CD44, as well as LYVE-1 (Lawrance et al., 2016; Lesley et al., 2004). Direct effect of FK506 on TSG-6, or effect of FK506 on interaction between TSG-6 and LYVE-1 or CD44 merits additional investigation.

**Fig. 7:**
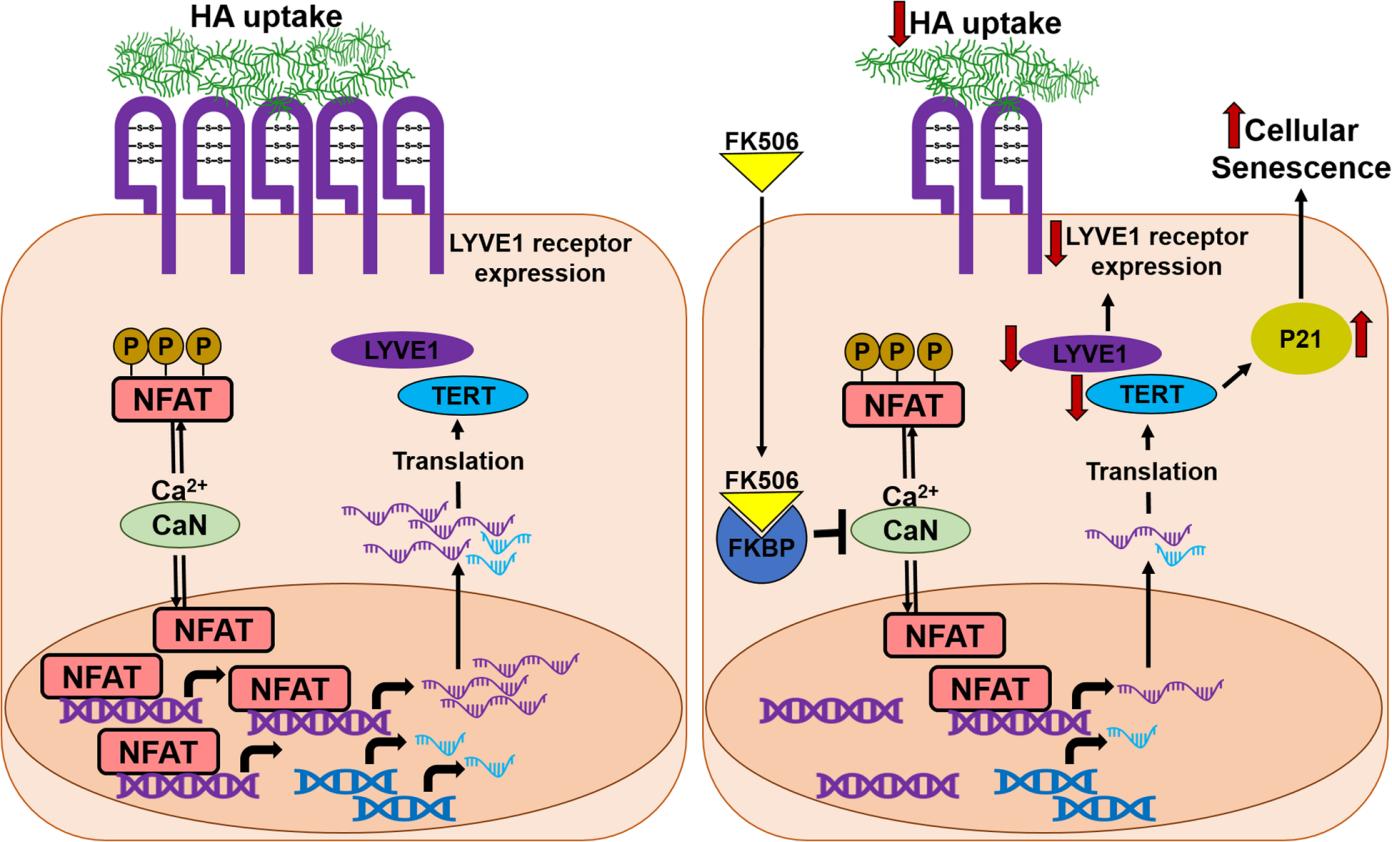
Schematic representation of FK506-induced lymphatic dysfunction. Calcineurin dephosphorylates NFAT proteins and induces translocation of NFAT into the nucleus (Hogan et al., 2003). This leads to increased transcription of NFAT target gene. Our study demonstrated that LYVE-1 is one of the target gene regulated by NFAT. This increases LVYE-1 receptor expression in lymphatic endothelial cells (LEC), where LYVE-1 plays an important role in HA-uptake and clearance. In LEC in vitro, FK506, a calcineurin inhibitor, prevents the nuclear translocation of NFAT, resulting in reduced LYVE-1 expression and, consequently, reduction in HA uptake. Additionally, FK506 treatment also reduces TERT expression, which results increased cellular senescence implicated by increased P21 expression in LEC

We have previously shown that stimulation of lymphangiogenesis alleviates acute lung allograft rejection (Cui et al., 2015); seemingly this improvement is mediated by LYVE-1-dependent HA drainage. Further, lymphatic deletion in a lung isograft results in tertiary lymphatic organ generation and airspace enlargement (Outtz Reed et al., 2019), highlighting the importance of the lymphatic vasculature in the health of lung allografts. Our data presented here show that, at least in vitro and ex-vivo, FK506 has deleterious effects on LECs (Fig. 7) due to its effect on cellular senescence, LYVE-1 expression, and reduced HA uptake. Treatment with FK506, however, should not affect cellular machinery required to produce and degrade HA. Also, unlike the accumulation of small-HA that has been shown to affect LEC cell proliferation and lymphangiogenesis in a LYVE-1-dependent manner (Bauer et al., 2018), the accumulation of exogenous high–molecular weight HA assessed in this study should not affect LYVE-1 activation.

How do we reconcile our findings with the known beneficial effects of calcineurin inhibitors as immunosuppressive therapies after lung transplantation? What are the implications of our findings-if any- for human lung transplantation?

There are some clues in the existing literature that our findings could indeed have some implications. First, there is significant accumulation of HA in chronic lung allograft rejection (Todd et al., 2014). Second, LYVE-1 depletion and reduced LYVE-1–HA interaction has been shown to prevent entry of immune cells into the lymphatics (Johnson et al., 2017; Mathur et al., 2006; Hodge et al., 2009), with resulting immune cell accumulation in allografts undergoing rejection (Baluk et al., 2009; Ji et al., 2014). These two findings should lead to increased lymphangiogenesis; however, in chronic lung allograft rejection (whether bronchiolitis obliterans or restrictive allograft dysfunction) there is no change in lymphatic vessel density (Traxler et al., 2017). We are tempted to hypothesize that chronic FK506 exposure could be responsible -at least in part- for this relative lymphatic deficiency, by inducing LEC senescence, decreasing LYVE-1 expression, and blunting inflammation-driven lymphangiogenesis. However, studies are needed to examine the long-term effects of FK506 exposure on LEC in vivo.

FK506 is the mainstay of immunosuppression in lung transplant with clear benefits on allograft rejection. However, despite similar immunosuppressive strategies, lung transplantation has the worse outcome of any solid organ transplant (Yusen et al., 2010; Valapour et al., 2018). There is a need for novel immunosuppressive therapies (Bharat, 2019; Hsiao et al., 2017). It is tempting to hypothesize that alternative strategies that could protect the lymphatic endothelium or enhance lymphangiogenesis (Stump et al., 2019) concurrent to current immunosuppressive strategies could protect lung lymphatics, enhance HA clearance, and result in improved outcomes in lung transplantation.

## Conclusion

Here we show that FK506, the mainstay of lung transplantation immunosuppression, alters lymphatic endothelial cell function, by inducing senescence, decreased LYVE-1 expression in vitro and ex-vivo in precision-cut-lung-slices with associated decreased HA-uptake in vitro (Fig. 7). The implications of our findings on human lung transplantation require further studies.

## Supplementary information

**Additional file 1: Supplementary Material and Methods** (Matys et al., 2006). **Supplementary Figure 1.** Effect of 48H treatment with FK506 on LYVE-1. Western blot analysis of LYVE-1 and β-actin proteins in lung lymphatic endothelial cells treated without and with FK506 (10 ng/mL and 15 ng/mL) for 48H. Molecular weight (kDa) for each protein is indicated on the right. Ratio of LYVE-1 to β-actin density was expressed as fold-change relative to control. Data represent mean ± SEM of three independent experiments, consisting of one technical replicate each. **Supplementary Figure 2.** FK506 prevents NFAT nuclear translocation. Lung lymphatic endothelial cells were treated as indicated. Samples of protein lysates (Entry: 27%), and nuclear (27%) fractions were separated by SDS-PAGE and transferred to nitrocellulose membranes, and reacted with antibodies against NFAT, PARP (nuclear marker) and GAPDH (cytoplasmic marker). FK506 resulted in a marked decrease in NFAT in nuclear fraction. Data represent mean ± SEM of 3 independent experiments (*p* < 0.05). **Supplementary Figure 3.** Effect of Cyclosporin A on TERT and LYVE-1 expression. Real-time PCR analysis of TERT (A) and LYVE-1 (B) mRNA in lung lymphatic endothelial cells treated with control or Cyclosporin A (10 μg/mL) for 48 h. Results were expressed as the fold change compared to control. Graphs represent the mean ± SE from three independent experiments. *p* < 0.05 (*) and *p* < 0.01 (**) by T-Test. **Supplementary Figure 4.** Effect of FK506 on other lymphatic markers. Real-time PCR analysis of podoplanin (PDPN) (A) and PROX1 (B) mRNA in control and FK506-treated (48 h) lung lymphatic endothelial cells. Results were expressed as fold change compared to control. Graphs represent the mean ± SE from three independent experiments. **Supplementary Figure 5.** Effects of LYVE-1 inhibition with function blocking antibodies on HA uptake in vitro and FK506 treatment on CD44 expression. LEC were plated in 6-well plates and treated with Isotype (control) and LYVE-1 monoclonal antibodies (10 μg/mL) for 72 h. Cells were then incubated in media containing 1000 μg/mL of FITC-HA for 5 h. Percentage of FITC-positive cells (A) were analyzed by flow cytometry. Real-time PCR analysis of CD44 (B) mRNA in control and FK506-treated (48 h) lung lymphatic endothelial cells. Results were expressed as the fold change compared to control. Graphs represent the mean ± SE from three independent experiments. **Supplementary Figure 6.** Western blot full images. **Supplementary Table 1.** Primary antibodies used in these studies. **Supplementary Table 2.** Real-time PCR primers.

## Data Availability

All data generated or analysed during this study are included in this published article [and its supplementary information files].

## Abbreviations

LEC: Lymphatic endothelial cells
TERT: Telomerase reverse transcriptase
LYVE-1: Lymphatic vessel endothelial HA receptor
CLAD: Chronic lung allograft dysfunction
NFAT: Nuclear factor of activated T-lymphocyte
TNF: Tumor necrosis factor
