# Supplementary material for: FK506 induces lung lymphatic endothelial cell senescence and downregulates LYVE-1 expression, with associated decreased hyaluronan uptake
Source: Mol Med. 2020 Jul 31;26:75. doi: 10.1186/s10020-020-00204-z (PMC7395348; doi:10.1186/s10020-020-00204-z)
Supplement: Supplementary file 1 — Additional file 1: Supplementary Material and Methods (Matys et al., 2006). Supplementary Figure 1. Effect of 48H treatment with FK506 on LYVE-1. Western blot analysis of LYVE-1 and β-actin proteins in lung lymphatic endothelial cells treated without and with FK506 (10 ng/mL and 15 ng/mL) for 48H. Molecular weight (kDa) for each protein is indicated on the right. Ratio of LYVE-1 to β-actin density was expressed as fold-change relative to control. Data represent mean ± SEM of three independent experiments, consisting of one technical replicate each. Supplementary Figure 2. FK506 prevents NFAT nuclear translocation. Lung lymphatic endothelial cells were treated as indicated. Samples of protein lysates (Entry: 27%), and nuclear (27%) fractions were separated by SDS-PAGE and transferred to nitrocellulose membranes, and reacted with antibodies against NFAT, PARP (nuclear marker) and GAPDH (cytoplasmic marker). FK506 resulted in a marked decrease in NFAT in nuclear fraction. Data represent mean ± SEM of 3 independent experiments (p < 0.05). Supplementary Figure 3. Effect of Cyclosporin A on TERT and LYVE-1 expression. Real-time PCR analysis of TERT (A) and LYVE-1 (B) mRNA in lung lymphatic endothelial cells treated with control or Cyclosporin A (10 μg/mL) for 48 h. Results were expressed as the fold change compared to control. Graphs represent the mean ± SE from three independent experiments. p < 0.05 (*) and p < 0.01 (**) by T-Test. Supplementary Figure 4. Effect of FK506 on other lymphatic markers. Real-time PCR analysis of podoplanin (PDPN) (A) and PROX1 (B) mRNA in control and FK506-treated (48 h) lung lymphatic endothelial cells. Results were expressed as fold change compared to control. Graphs represent the mean ± SE from three independent experiments. Supplementary Figure 5. Effects of LYVE-1 inhibition with function blocking antibodies on HA uptake in vitro and FK506 treatment on CD44 expression. LEC were plated in 6-well plates and treated with Isotype (control) [file 10020_2020_204_MOESM1_ESM.docx]

**Supplementary information**

**Supplementary Material and Methods**

**Cell fractionation and immunoblotting**

LEC were seeded at a density of 2x10^6^ cells per 60 mm culture dish. Cells were serum-starved for 6 hours and then treated with 10 ng/mL and 15 ng/mL of FK506 in full media. After 72 hours, cells were washed with sterile PBS and protein lysates (nuclear fraction and whole-cell lysates) were harvested. Nuclear protein extracts were prepared using CelLytic Nuclear Extraction Kit (Millipore Sigma, St. Louis, MO) according to the manufacturer’s protocol. Whole cells lysates were prepared using RIPA lysis buffer supplemented with protease and phosphatase inhibitors.

To examine NFAT-1 nuclear translocation, equal fractions of nuclear and total protein were separated on NuPAGE Bis-Tris 4-12% gels (Thermo Fisher Scientific, Waltham, MA) and transferred to a polyvinylidene fluoride membrane, which was then exposed to the indicated primary antibodies overnight (Supplementary Table 1). To examine the effects of FK506 on other indicated proteins, 30 µg of whole-cell lysates were subjected to sodium dodecyl sulfate-polyacrylamide electrophoresis, followed by transfer to a polyvinylidene fluoride membrane, which was then blocked and incubated with the indicated antibodies (Supplementary Table 1) overnight at 4°C.

Proteins were then detected with HRP-conjugated goat anti-rabbit or anti-mouse IgG (H+L) antibodies as necessary (1:1000, Thermo Fisher Scientific, Waltham, MA), and visualized using SuperSignal chemiluminescent substrate (Thermo Fisher Scientific, Waltham, MA). Band density for each protein was visualized and analyzed using Gel Doc XR+ System and Image Lab 6.0.1 (Biorad, Hercules, CA).

**Immunofluorescence**

LEC were seeded in a 4-well chamber slide (Falcon, Big Flats, New York) and treated as indicated for 72 hours. Cells were then fixed with 4% paraformaldehyde for 15 min and blocked with PBS-Triton X-100 (0.3%) and 5% normal goat serum for 1 hour at room temperature. Cells were then incubated with anti-P21 antibody overnight at 4°C, followed by secondary antibody staining for 1 hour at room temperature. Slides were then mounted with mounting medium containing 4,6-diamidino-2-phenylindole-2-HCl (Vector Laboratories Inc, Burlingame, CA). Nine to 12 images were collected per condition per biological replicate using an Olympus FluoView FV-10i confocal laser scanning microscope (Olympus, Tokyo, Japan) and analyzed using ImageJ image analysis software (National Institutes of Health, Bethesda, MD) (Intensity Ratio Nuclei Cytoplasm Tool <http://dev.mri.cnrs.fr/projects/imagej-macros/wiki/Intensity_Ratio_Nuclei_Cytoplasm_Tool>, National Institutes of Health, Bethesda, MD)

**Reverse transcriptase and PCR assay**

Total RNA was extracted from LEC lysates after 48 hours of treatment with FK506 using the RNeasy mini kit (Qiagen Cat#74104, Germantown, MD), as per manufacturer’s instructions. One microgram of RNA was reverse transcribed into cDNA using AmplifiRivert Reverse transcription kit (GenDEPOT Cat#R5101, Katy, TX), as per manufacturer’s instructions. Quantitative PCR was then performed using RT2 SYBR Green qPCR master mix (Qiagen, Cat # 330529) on an Applied Biosystem StepOnePlus Real-Time PCR System (Thermo Fisher Scientific, MA). All primers were synthesized by Sigma (Sigma Aldrich, Saint Louis, MO) and are listed in Supplementary Table 2.

**Generation of LYVE-1 promoter constructs**

LYVE-1 promoter sequence upstream of the transcription start site was analyzed for the presence of NFATc binding sequences (5’-ggaaaa-3’ or 3’-ttttcc-5’) using TRANSFAC database (58). The LYVE-1 promoter fragment (-124 bp to +125 bp) upstream of transcription start site consisting of one NFATc binding site (ttttcc) was amplified using primers: Forward - 5'-gcccggagctcgactccacaggcagtaacagt-3’ and Reverse: 5’-tccccccggggagccagggaaacacctcaga-3’. The fragment was digested and cloned between NheI (5’) and HindIII (3’) restriction sites within the multiple cloning site of digested pGL3_Basic vector (Promega Cat# E1751, Madison, WI) creating LYVE-1 promoter-luciferase constructs, labeled as P-124/+124. Further, site-directed mutagenesis was performed using Phusion High Fidelity DNA polymerase (ThermoFisher Scientific, MA) to create a mutant P-124/+124 construct with a one-base pair mutation in the NFATc binding site using primers: Forward - 5’-gcattagctaaattgtccagaaggc-3’ and Reverse: 5’-tgactcagaaaacacacaaatgaggaaggc-3’. All constructs were confirmed for correct sequence by Sanger sequencing using primers: 5'-ctagcaaaataggctgtccc-3', 5'-ggagagcaactgcataagg-3', and 5'-gatgagtttggacaaaccac-3'.

**Supplementary Figures**


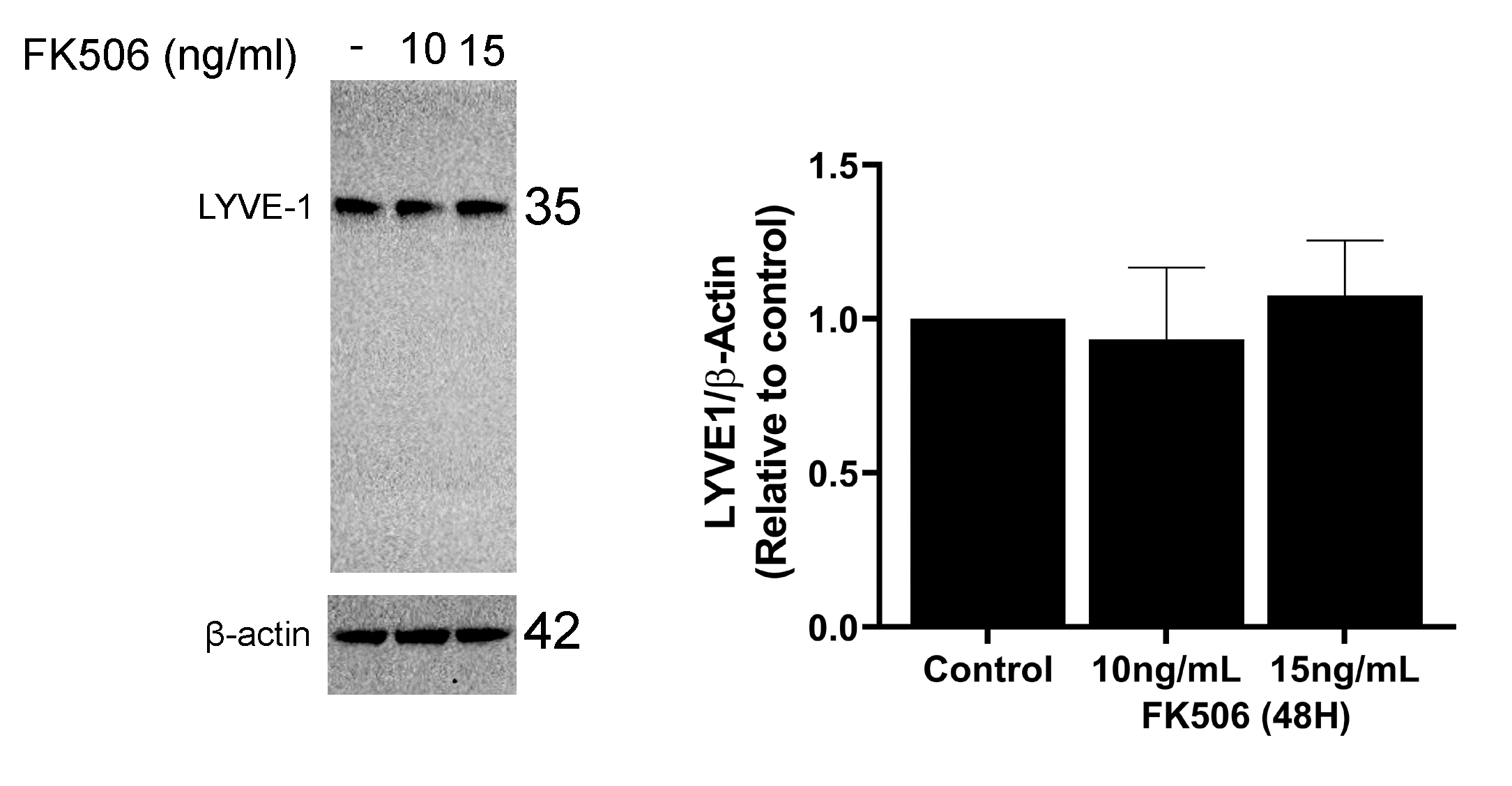


**Supplementary Figure 1. Effect of 48H treatment with FK506 on LYVE-1**. Western blot analysis of LYVE-1 and β-actin proteins in lung lymphatic endothelial cells treated without and with FK506 (10ng/mL and 15ng/mL) for 48H. Molecular weight (kDa) for each protein is indicated on the right. Ratio of LYVE-1 to β-actin density was expressed as fold-change relative to control. Data represent mean ± SEM of three independent experiments, consisting of one technical replicate each.

**
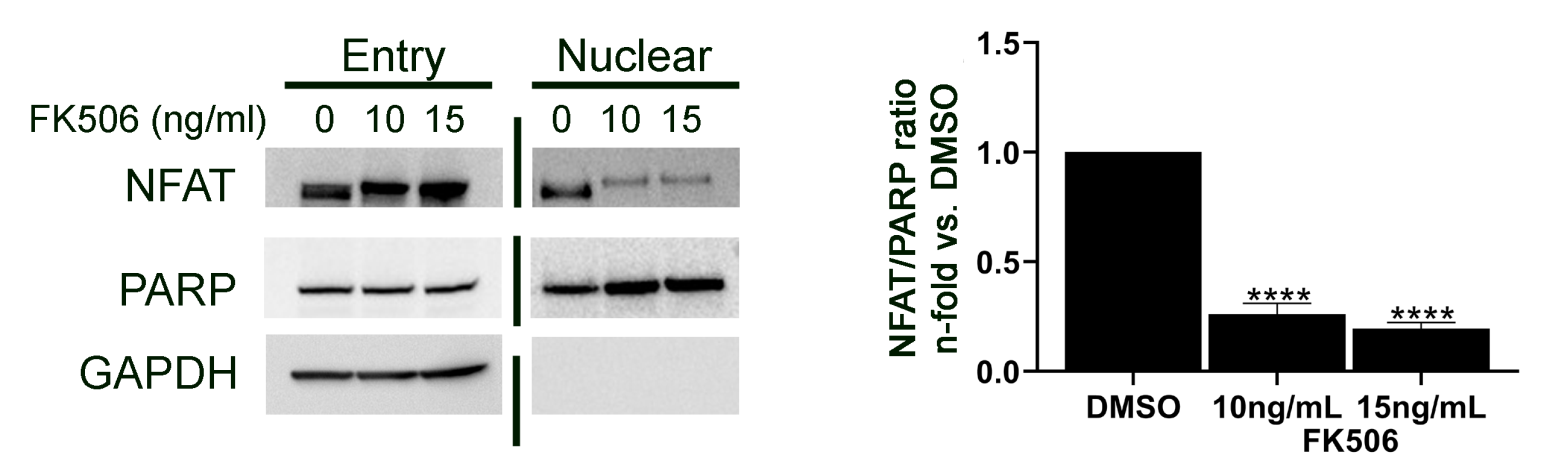
**

**Supplementary Figure 2. FK506 prevents NFAT nuclear translocation.** Lung lymphatic endothelial cells were treated as indicated. Samples of protein lysates (Entry: 27%), and nuclear (27%) fractions were separated by SDS-PAGE and transferred to nitrocellulose membranes, and reacted with antibodies against NFAT, PARP (nuclear marker) and GAPDH (cytoplasmic marker). FK506 resulted in a marked decrease in NFAT in nuclear fraction. Data represent mean ± SEM of 3 independent experiments (p<0.05)

**
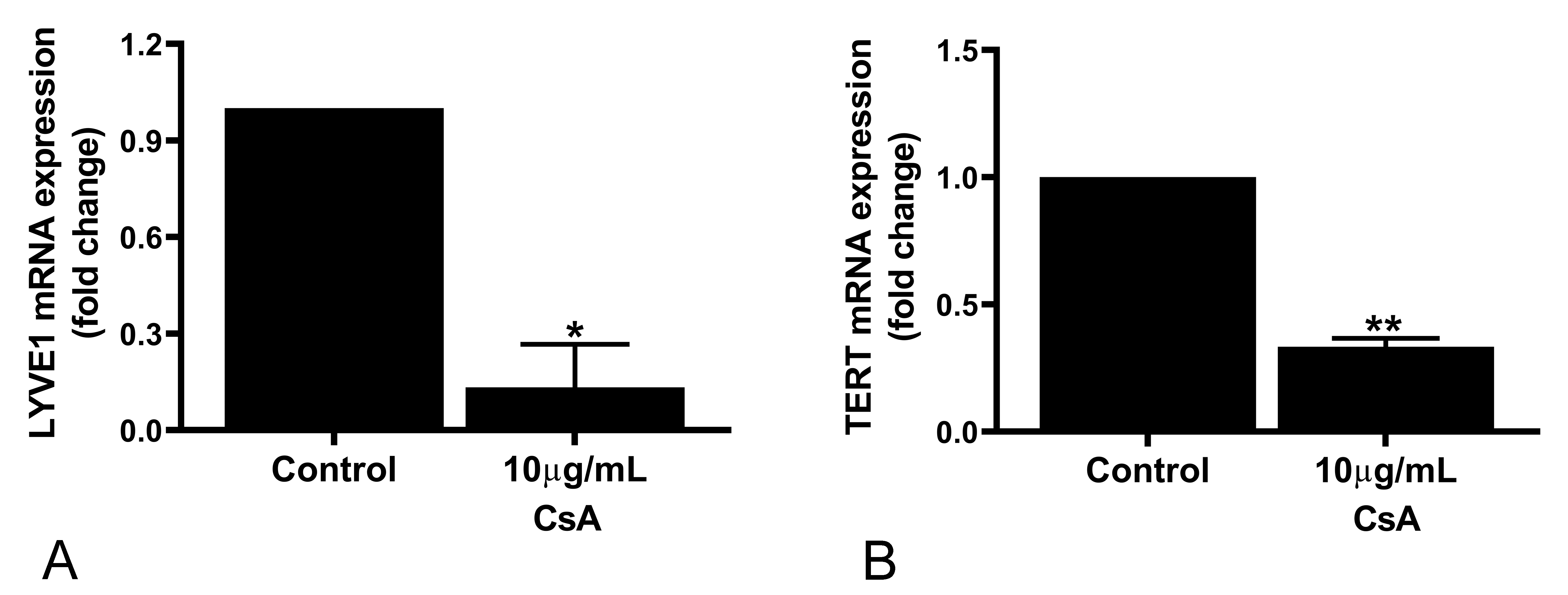
Supplementary Figure 3. Effect of Cyclosporin A on TERT and LYVE-1 expression.** Real-time PCR analysis of TERT (A) and LYVE-1 (B) mRNA in lung lymphatic endothelial cells treated with control or Cyclosporin A (10µg/mL) for 48h. Results were expressed as the fold change compared to control. Graphs represent the mean ± SE from three independent experiments. p<0.05 (*) and p<0.01 (**) by T-Test.


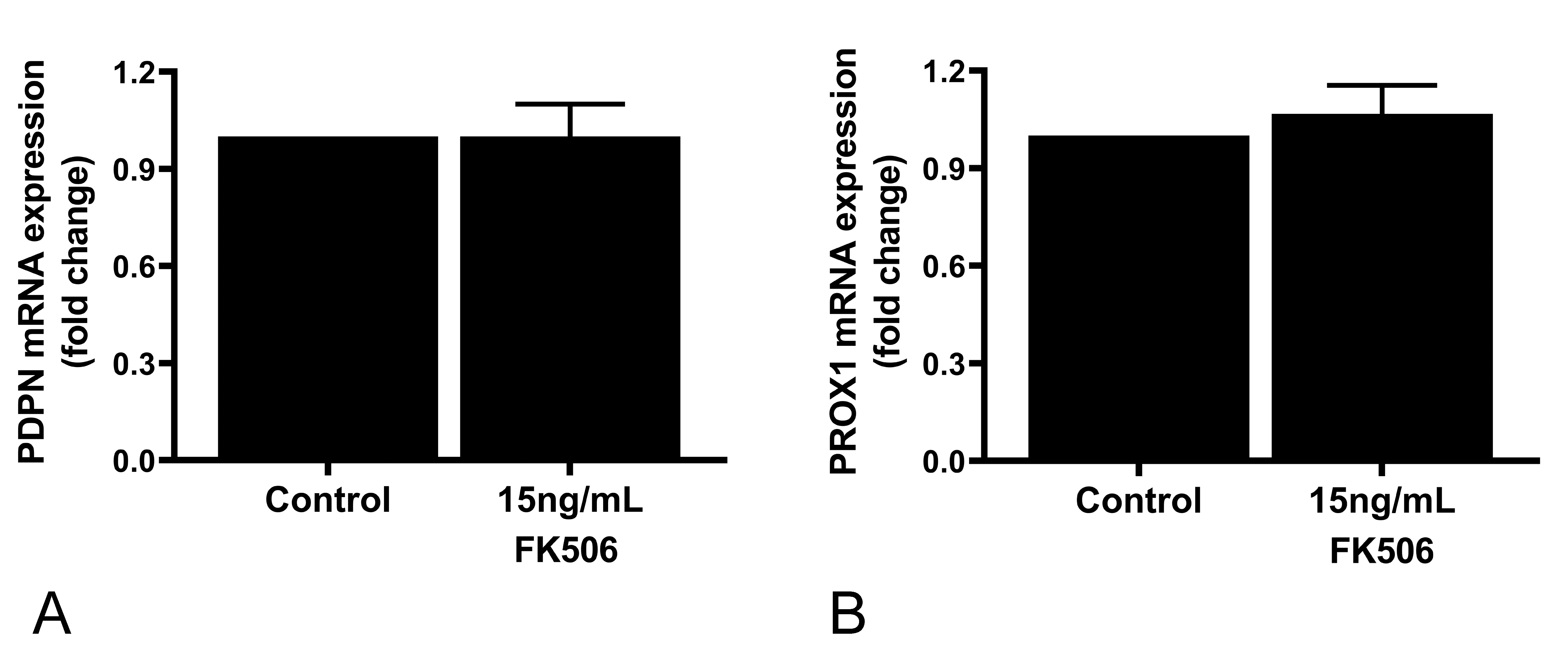


**Supplementary Figure 4. Effect of FK506 on other lymphatic markers.** Real-time PCR analysis of podoplanin (PDPN) (A) and PROX1 (B) mRNA in control and FK506-treated (48 hours) lung lymphatic endothelial cells. Results were expressed as fold change compared to control. Graphs represent the mean ± SE from three independent experiments.

**
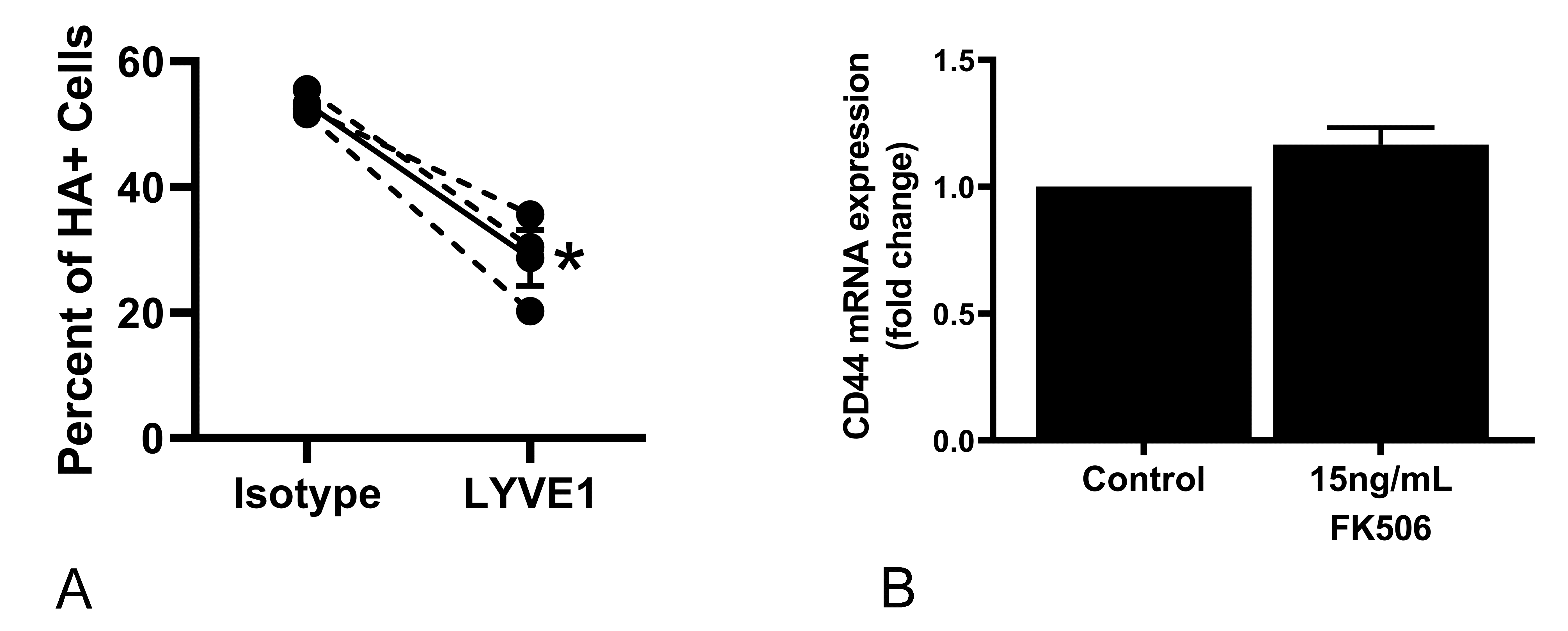
Supplementary Figure 5.: Effects of LYVE-1 inhibition with function blocking antibodies on HA uptake *in vitro* and FK506 treatment on CD44 expression.** LEC were plated in 6-well plates and treated with Isotype (control) and LYVE-1 monoclonal antibodies (10 µg/mL) for 72h. Cells were then incubated in media containing 1000 µg/mL of FITC-HA for 5h. Percentage of FITC-positive cells (A) were analyzed by flow cytometry. Real-time PCR analysis of CD44 (B) mRNA in control and FK506-treated (48 hours) lung lymphatic endothelial cells. Results were expressed as the fold change compared to control. Graphs represent the mean ± SE from three independent experiments.

**Supplementary Figure 6. Western blot full images**

**
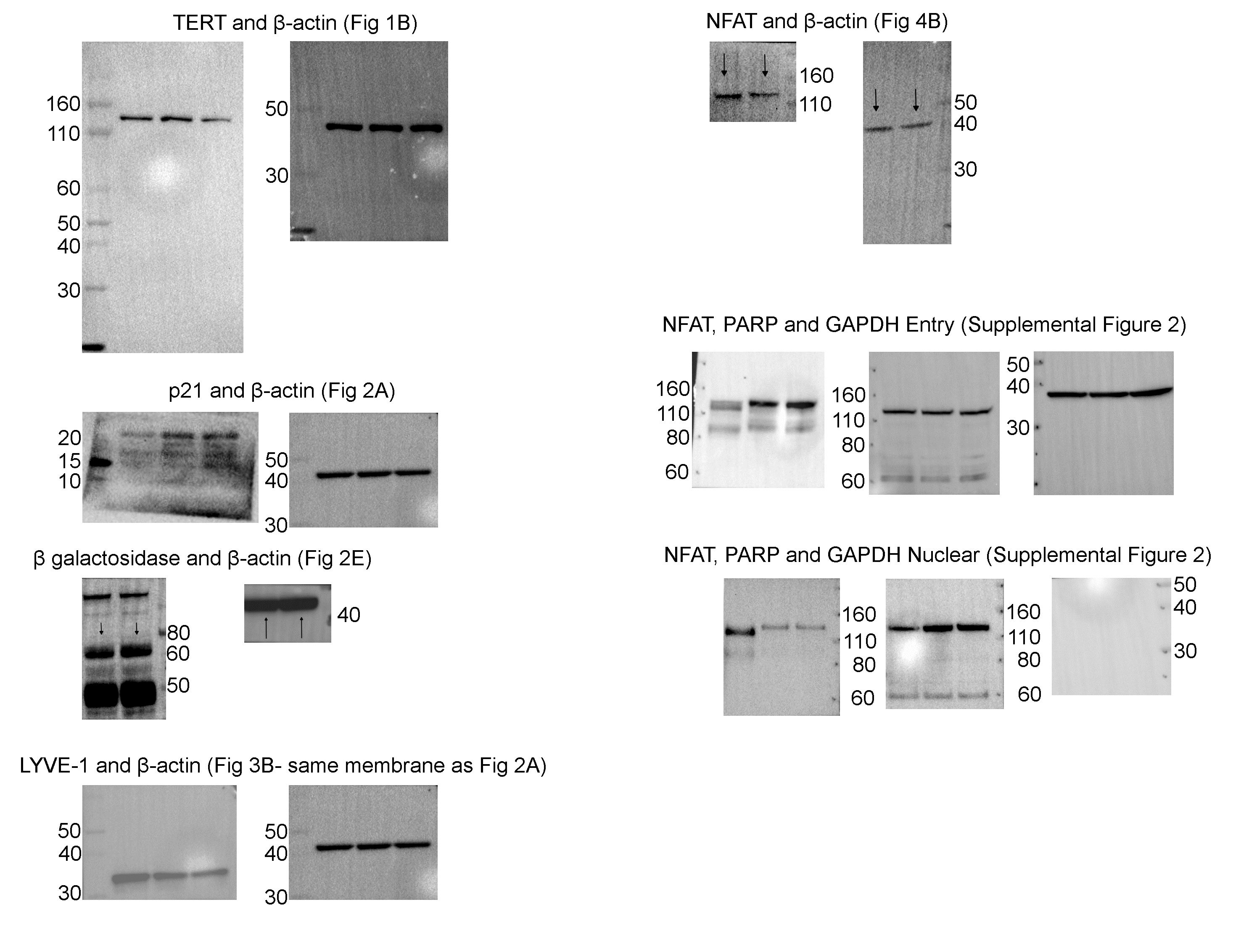
**

**Supplementary Table 1: Primary antibodies used in these studies**

| **Antigen** | **Source** |
| --- | --- |
| TERT | Abcam; Catalog number ab32020 |
| LYVE-1 (WB) | Abcam; Catalog number ab183501 |
| LYVE-1 (Flow) | Thermo Fisher Scientific; Cat# 25-0443-80 |
| LYVE-1 (Function blocking) | R&D System; Cat#MAB20892 |
| Isotype control | R&D System; Cat#MAB002 |
| NFAT-1 | Cell signaling technology; Catalog number 5861, clone D43B1 |
| P21 | LSBio; Catalog number LS-C136937 |
| GAPDH | Cell signaling technology; Catalog number 5174, clone D16H11 |
| β-Galactosidase | cell signaling technology; Catalog number 27198 |
| β-Actin | Santa-Cruz; Catalog number sc-47778 |

**Supplementary Table 2: Real-time PCR primers**

| **Target gene/accession number** | **Primer sequence 5’ to 3’** |
| --- | --- |
| Human TERT (exon7)/NG_009265.1 | Sense: GGAGTCCCAGGTGTGTCTGTA  Antisense: CAAGGCACACAGCTCATCAT |
| Human Lyve-1/NM_006691.4 | Sense: TGTCAAAAGGTATGTGAAGG  Antisense: TCTAAACTTCAGCTTCCAGG |
| Human β2M/NM_004048.4 | Sense: AAGGACTGGTCTTTCTATCTC  Antisense: GATCCCACTTAACTATCTTGG |
| Human CD44/NM_000610 | Sense: CCAGAAGGAACAGTGGTTTGGC  Antisense: ACTGTCCTCTGGGCTTGGTGTT |
